# Monthly Summary

**Published:** 1893-09

**Authors:** 


### Monthly Summary.

BANDING ROOTS.—There is no doubt—in theory—about the desirability of banding a root you are going to crown. But then in practice you find some reasons that operate to favor the crown without a band. One objection to putting a band on a root is that, as frequently done, the band is allowed to impinge on the dental ligament, which, being so plentifully supplied with nerves, is very likely to induce inflammation of a serious character and endanger the life of the tooth. I recently extracted a bicuspid root with a crown on from the mouth of a dentist, because the band had been permitted to extend as far as the alveolar process, setting up sufficient inflammation, during the period of a year or so to make it advisable to extract on account of loss of tissue by absorption. It might be proper to notice here that this failure did not occur because the root was banded, but because it was not *properly* banded, although the operator who fears to band a root for the reason that he may not perform it perfectly, gives a good and sufficient reason for not doing so—the same that he would give for putting an air chamber in a plate after he satisfies himself that after all an air chamber only covers the imperfections of his work—only in so far as he is himself concerned.

The increased difficulty of setting a crown with a band has contributed to its unpopularity, as there is no convenient escape for the surplus cement, and the greater number of its points of bearing tends to produce rocking and a consequent lack of stability when set. A patient who had two crowns set by the same dentist, one with a band and one without, said he would not have another crown inserted with a band; that the band crown was very painful to fit and adjust and it appeared to be somewhat loose, or at least produced continued, slight irritation and had an uncomfortable feeling. This is not a scientific objection but it may be a practical one. The truth is that a banded crown is the best, granting that the work is perfectly done, and the principal reason why all roots are not banded is that an all porcelain crown very often makes a serviceable operation, and the inability of the operator to successfully make and adjust a banded crown as he knows it should be done.—*Pacific Dental Journal.*

---

MUTILATION OF THE TEETH BY SAVAGES.—Abstract by the "Odontographic Journal's" Translator.—Dr. Magitot has recently published in Paris an interesting account of the mutilation of the teeth practiced by various savage races. One sort, frequently encountered upon the African coast, and the west coast of New Guinea, consists in breaking off part of the incisors by means of a knife and a piece of wood, and is performed between the ages of 20 and 25.

The custom of extracting both central incisors, is met with in both hemispheres. According to Zerate, the Peruvians have done this from time immemorial, as a mark of slavery set upon conquered tribes. In Africa it is observed on the Congo, among the Hottentots and the Batoxas.

Mutilation by filing has its especial centre in the Malay Archipelago, whence it has extended to neighboring islands. Among the Mohammedans this is a religious act performed with great solemnity at the age of puberty. The sort and manner of the filing depends upon the family and caste. The operation is accomplished by an expert, called the *Tukang Pangar* or filer, who uses a chisel, three tiles, two files, a saw and a pair of pinchers, rubbed with arsenic and lemon juice.

Among certain tribes on the Senegal, the upper temporary incisors of all girls are extracted, producing a projection of the lower jaw, so that the under lip overlaps the upper.

In Indo-China and Japan the young women color the teeth at marriage with black lacquer. This process requires much time and money, hence is confined to the well to do classes.

Livingstone noted that among the Kaffirs a child whose upper teeth erupt before the lower, is regarded as a monster and is killed.

On the upper Nile the blacks have their upper teeth extracted through fear of slavery, this injuring their market value. As Abbee Peritat writes, there exists in some adjacent tribes among the Esquimaux, the custom of dividing the upper teeth crosswise. This is founded on a local tradition, and the reason given is that it is to prevent the human chin from resembling that of the dog.—*Odontographic Journal.*

---

ROOT TREATMENT.—By E. L. Clifford.—If we use eucalyptol we have an agent that is more penetrating, powerful and clean, and certainly of a much more pleasant odor. If we could get the oil of eucalyptus which is made by Sanders & Sons, of Australia, it would be the thing for us to use. As you well know, there are thirty or forty different species of eucalyptus. There are four special ones that are used, and Dr. Black has shown from some bacteriological experiments he has made that the power of eucalyptol is certainly greater than the oil of eucalyptus; in fact, oil of eucalyptus appears on a par with almost all the other antiseptics that have been experimented with. We know also from our researches that a good many of our antiseptic theories have been over-stated. We have learned from them that it is not necessary in order to kill a micro-organism to put salt on its tail or strike it in the head. If we remove any one of the four elements that are absolutely necessary for its growth, we can prevent its future growth. The day of rational therapeutics has not arrived, and we cannot throw away our empirical methods simply because we do not know how they act. If we did, we would not put arsenic in a tooth to-day. We do not know how it kills a pulp. The *rationale* of its action has never been demonstrated sufficiently so that the majority of the profession could accept it. In order that he may be doubly sure, the operator floods his cavities with chloroform or ether prior to the application of these oils. As the doctor says, there may be extraneous matter there that abounds in the oils and fats that heat does not mummify. These oils and fats are freed and absorbed to a great extent by the tubuli of the teeth. With ether we can dissolve the oils and fats. With chloroform we can place the surrounding tissues continguous to the parts in an anæsthetic condition; consequently we can make these operations much less painful. Eucalyptus or eucalyptol, or any other agent, will mummify with the assistance of heat, and I believe, as I said two or three years ago, that when the apical foramen is thoroughly sealed with an indestructible substance, freedom from future trouble is just as sure as if you had extracted the tooth.—*Dental Cosmos.*

---

UNFERMENTED BREAD FOR INVALIDS.—Undone bread has had the reputation of being unhealthful for countless generations, but fermented bread *well baked* has received no unsavory reputation.

Having no desire to gain notoriety or alarming the common people by calling their attention to matters not within their power to remedy, yet I wish to show some reasons why unfermented bread is decidedly more wholesome and nutritious than any form of fermented bread.

Bread made with yeast goes through the long process of raising and kneading. In many homes the bread is kneaded and raised there.

In homes, as well as in bread-making establishments, the dough is often sour before it is ready to bake. Either carelessness or lapse of time, or some other condition which favors a fermenting process, makes the bread non-nutritious without a remedy. Bi-carb soda is added to sweeten the rotted starch, and the loaves are baked and put before growing children and adults as the *staff of life.*

It must be apparent to all that the long process of bread-making gives a chance for the gathering in of germs. The longer any substance which is to be eaten is expossd, the greater the chance that germs may find a habitation.

Besides the danger from long exposure of yeast bread to the air, fermentation uses up a large portion of the nutrient elements of the bread, and the loss is in proportion to the time given to the process.

If yeast bread is eaten it should be carefully compounded, closely watched, well covered, during the process of raising, and well baked as soon as possible after the raising has commenced.

Breat is thought to be more wbolesome by toasting. This adds another barrier against the fermenting process after it is taken into the stomach. All the conditions to produce a ferment are present in the stomach when yeast bread enters. Until the integrity of the stomach is unimpaired and vital changes commence quickly, fermentation of the bread takes place and all other substances taken are unfitted for nutrition.

Bread raised quickly from chemicals introduced into the flour and water makes the most healthful bread for all classes. The chemicals, cream of tartar and soda, are perfectly harmless. The baking powders are supposed to be compounded of these two chemicals, and in such proportions as to form carbonic gas when subjected to moisture and heat.

Clean water, salt and flour, of any kind, with a spoonful of baking powder, make light wholesome bread. The less the break is worked the better it is. This quick mode of raising bread is the most perfect method conceivable. There is no chance for germs to enter the dough. The bread is sweet, no time given for it to sour; it will keep longer after it is baked and remain unfermented in the stomach a much longer period than any other bread. When gastric and other alimentary fluids are scanty in quantity and defective in quality, as they are in most invalids, great care should be taken that grain products are not in a state to set up a ferment instead of being digested —E. M. ROYS-GAVITT in *Woman's Medical Journal.*

LOCAL ANÆSTHETIC NOSTRUMS.—On this subject in the May "Cosmos" Dr. E. C. Kirk flashes some original light in the way of research into the dim corners of "made-to-sell" anæsthetics. Having purchased samples in the open market through a third party of several of the most generally used preparations, he sent them to Samuel P. Sadtler, the professor of chemistry of the Philadelphia College of Pharmacy, a gentleman of reliable information for accurate work, for an analysis of their principal ingredients and with particular reference to the amount of cocaine they contained. Notwithstanding the assertions almost uniformly made by the manufacturers, that they contained no cocaine, "may be used on all classes of patients, the young, the old, the sick, the healthy," etc., including a positive assurance of no after effects, chemical analysis showed the following results.

| | | | | |
|---|---|---|---|---|
| Dickson's . . . | 3.90 | per cent. | of Anhydrous | Cocain. |
| Arophene . . . | 1.46 | " | " | " |
| Jessop's . . . | 2.63 | " | " | " |
| Dorsenia . . . | 0 20 | " | " | " |
| Weinman's . . | 5.68 | " | " | " |
| Odontunder . . | 1.35 | " | " | " |
| Dental Surprise . | 1.46 | " | " | " |
| Barr's . . . . | none | " | " | " |
| Eureka . . . . | 3.26 | " | " | " |
| Anestheto-Obt'dent | 3.39 | " | " | " |

Barr's being the only one that does not show cocaine. The Arophene Mfg. Co. have hastened to reply to the article, saying that it has done them a great injustice for the reason that the analysis is wrong—their preparation does not contain the amount of cocaine stated, but contains less than one per cent., and less than one per cent. has never produced any toxic symptoms—a defense that does not defend.

The proprietors of Dorsenia have furnished the "Pacific Dental Journal" with the formula of their preparation, which is as follows: "Dorsenia is the essential anæsthetic and antiseptic constituents of Macropiper Methysticum, Mentha Arvenis, Baptisia, Eucalyptus, Camphor and Phenol in combination." This latter preparation is being sold now as "non-secret and professional." The offensive odor arising from these secret anæsthetics, as soon as their true constituents are shown up, is very pungent, to say the least, keeping in mind the grossly misleading, not to say untrue, statements of their proprietors concerning their harmlessness.—*Pacific Dental Journal.*

---

CARCINOMA OF THE MOUTH.—By R. F. Weir.—We have here in this man an interesting case to show you. He is forty-five years old, and has had trouble with his mouth for some fifteen or seventeen months. He has not suffered much pain, has not lost flesh or strength, and his speech is not very much interferred with. What does this mean? It means that the mobility of the tongue has not been impaired, that there is no infiltration into the substance of the muscle; and you will find in all conditions, whether syphilitic or carcinomatous, that the patient soon begins to talk in an abnormal manner.

I think you can all see a whitish mass the size of a half dollar, a little elongated, with numerous divisions on its surface. This condition looks like a papilloma, and its base of attachment to the tongue is not as large as the surface of the tumor. As I feel along the tongue in this way, I elicit no pain, and usually carcinomatous growths are quite painful, I find along the top of the tongue a number of changes showing that he has had damage done to his mucous membrane there. Furthermore, on passing one finger into his mouth and another finger outside under the jaw, I find one large but very soft mass. It seems as if this mass were an enlarged sub-maxillary gland not the ordinary gland over the sub-maxillary. The very softness of this enlargement gives me a clue to the trouble.

Now, the question comes up, is this a papillomatous growth? These growths, as a rule, are harmless when small, but we must always remember that a papillomatous growth may occur with other growths that are quite serious in character, and notably in connection with a carcinomatous growth. We are more apt to find papillomatous growths on mucous surfaces where there is a greater possibility of the growth being undisturbed, and hence we have them in the bladder, rectum and mouth. All the more likely are we to have these growths where the mucous layers are very thick and papillæ are present. This obtains markedly in the tongue. I believe we have here a trouble that is of an extremely dangerous and suspicious character, viz.: a carcinoma. It is, indeed, an unusual thing to find this kind of growth present in the mouth. I have seen it numerous times present about the anus.

I ask, then, that you remember but two facts in connection with this case; the first is the peculiar color and appearance of the papillomatous growth, and second the absence of infiltration, which may also be present.

You can hope for a more favorable prognosis in this kind of malignant growth than in any other. This is due to the fact that there is no infiltration, and owing to the absence of this infiltration, you can feel when you cut wide of the growth that you are in healthy tissues.—*International Journal of Surgery.*

---

DENTAL DOTS DISTILLED.—By D. V. Beacock, Brockville, Ont.—Ethylate of sodium is good to use on hypertrophied gums, free from pain and danger even if used in excess.

Cocaine dissolved in chloroform, one grain to one-eighth ounce of chloroform, is good to extirpate pulps without pain.

Take a small-sized sewing needle; at the distance of, say, three-quarters of an inch from the point, bend into the form of an S, the point of the needle forming the long leg, useful in filling labial cavities under the gum; stick the point into the neck of the tooth below the rubber dam, just above the edge of the cavity, lift the upper edge of dam over the eye end of the needle, and the resiliency of the rubber will keep the needle in place and the cavity dry. It is far ahead of any clamp for the above purpose. To prevent the eye of the needle penetrating the dam, put a little bead of shellac on the end.

